# Optimizing palliative care and support for pets –perspectives of the pet-parent and the veterinarian

**DOI:** 10.3389/fvets.2023.1162269

**Published:** 2023-05-19

**Authors:** Wendy W. T. Lam, Richard Fielding, Lok Yi Choi

**Affiliations:** ^1^Jockey Club Institute of Cancer Care, LKS Faculty of Medicine, The University of Hong Kong, Hong Kong, SAR China; ^2^School of Public Health, LKS Faculty of Medicine, The University of Hong Kong, Hong Kong, SAR China; ^3^Tung Chung Animal Clinic, Hong Kong, SAR China

**Keywords:** palliative care, communication, supportive care, end of life care, psycho-oncological care

## Abstract

As animals benefit from improved chronic disease care, more pet-parents and veterinarians face issues of late life and terminal care. Management of life limiting disease commonly considers the timing of euthanasia, often overlooking the role of supportive palliative care. Necessary communications between vet and pet-parents are rarely emphasized. However, as in human palliative care, the central role of good communications is critical. In particular, three communication elements are primary, namely: empathic communication and shared decision-making; managing progressive symptoms, and; advanced directives. Moreover, focusing only on euthanasia can easily discount the profound emotional legacy of bereavement. This Perspective illustrates how communications policies derived from human palliative care are exemplified in the management of a case of canine lung cancer, to the wider practice benefits of pets, pet-parents and veterinary practice staff.

## 1. Methods

This single-case study derives from participant observation data-gathering from the first and second author's own pet-parent experience of managing the terminal illness of their toy Poodle, Joe, who died from a primary pulmonary adenocarcinoma. All authors were approached and verbal and written informed consent was sought, and confirmed by their willingness to write this paper. The pet's verbal consent was sought by proxy, from the pet-parents. Clinical case data was extracted from the patient's medical record by a qualified veterinarian, the main care provider.

## 2. Introduction

Advances in veterinary diagnostics and treatments have increased geriatric companion animal numbers and management decisions around end-of-life issues. Organ failure is the main incurable life-limiting condition of older dogs and cats, many suffering the same diseases as their humans. Cancer rates however, are lower. U.S. insurance industry data indicate overall rates of cancers in pedigree dogs around <3%, but in some breeds may be considerably higher (1, 2) though such data are only indicative. Soft tissue, skin, bone and hematological cancers predominate (2, 3). As such, pet-parents increasingly face the challenges of caring for aging pets with life-limiting disease. As with human patients, personalized palliative care plays an essential role to minimize suffering in pets with incurable and progressive disease. Most animal palliative care literature focuses on managing the pet near the end-of-life and on euthanasia (4). For people, palliative care, by definition, is care that aims to maximize quality of life during life-limiting disease by effective symptom management, establishing clear care goals, maximizing independence and offering psychosocial support (5). We believe this definition is also applicable in veterinary care. This Perspective examines palliative care, integrating pet-parent and veterinarian views on the process of optimizing palliative care for companion animals.

## 3. Case

Joe, a neutered male brown toy poodle, was blind, overweight with Inflammatory bowel disease and only a few teeth when “rescued” aged 10-years (2015). Fiercely independent, he quickly navigated complex daily walks, needing guidance only on steps. Over time, Joe's Vet (LYC) managed his Calcium Oxalate urolithiasis (cystotomy, 2017), hip subluxation (osteotomy, 2018), and heart murmur (Grade IV/VI). A carefully-balanced home-cooked diet helped renormalized his weight. Bi-annual blood profiles indicated persistent borderline/high Calcium^+^ and ALKP (2017), unmodified by restricting Oxalate intake, remaining enigmatic despite exhaustive investigations. In Spring, 2020 a mild persistent cough and hesitancy/panting during walks emerged. Echo and X-ray (May 2020) revealed mild diffuse interstitial patterns in caudal lung fields. By early March 2021, caudal lung field crackles were apparent and a thoracic x-ray was suggestive of lung consolidation/dysplasia. Referral to a palliative care Internist for small animals, for FNA, CT and Ultrasound investigation confirmed a right caudal lung mass (7.4 × 5.9 × 5.3 cm), probably primary Pulmonary Adenocarcinoma, displacing both the bronchus and Aorta. After a surgical consult, the Internist suggested pneumonectomy, which, if successful, offered potential life expectancy of up to 12 months.

Given Joe's age, tumor mass, and likely operative trauma, the pet-parents chose palliative care under his usual Vet. Joe survived comfortably until early August 2021 when he was euthanized at home, age 16, due to rapidly-escalating respiratory distress, using intravenous infusion of Sodium Pentabarbital 200 mg/ml at a dose of 200 mg/kg.

## 4. The pet-parent perspective

This personal experience emphasized three important aspects in optimizing palliative care for pets and companion animals, namely empathic communication and shared decision-making, managing progressive symptoms, and advanced directive.

### 4.1. Empathic communications and shared decision-making

Empathic communications underpin all good health care for humans and animals. Providing the opportunity to discuss with the pet-parent the diagnosis and prognosis *in a compassionate manner*, 1. builds a good, trusting relationship between the pet-parent and veterinarian, thereby 2. facilitating decision-making for effective management by the veterinarian. Empathic communication includes delivering the diagnosis in a sensitive manner, acknowledging the likely emotional impact this has by using empathic statements.

#### 4.1.1. Breaking bad news

An old dog inevitably prompts end-of-life awareness, but it remains a shock for the pet-parent to be told that time is imminent. Joe's Vet titrated diagnostic information in response to Joe's pet-parents' emotional reactions, pausing frequently to allow assimilation, before moving on to management options. A strategy for this is as follows: First, ask how much pet-parents want to know about the diagnosis and prognosis and inform accordingly. Break bad news using an Assess; Disclose; Assimilate (ADA) approach (6): *Assess* pet-parent understanding. This reveals what they need to be told. *Disclose* sensitively, pausing to allow questions and emotional adjustment. Then help the pet-parent *Assimilate* the information. Use of lay terms and encouragement helped Joe's pet-parent's accept both diagnosis and prognosis. ADA is not a one-off exercise, but a tool for repeated use across the illness (6).

#### 4.1.2. Clarifying goals

During the diagnostic consultation, available treatment options and associated risks and benefits were outlined, including mentioning possible positive outcomes, to allow retention of some realistic hope. We shared our goals for Joe: to maximize his comfort and independence at home for as long as possible. Pet-parents' preference for life-sustaining treatments are likely influenced by expectations of treatment outcomes, so ask pet-parents about their outcome priorities and expectations. These often depend on how different outcomes are framed. Recommending surgery required emphasizing survival duration, but QoL (and financial!) costs likely dictate many pet-parents' choices. Pneumonectomy is traumatic, painful, and would have severely impaired Joe's independence, if he survived the operation and hospitalization stress. Without surgery, Joe's expected prognosis was 6 months. Palliative care would provide at least equivalent good QoL duration. For many pet-parents, underemphasizing surgical downsides when recommending treatment could skew decision-making. Clear information about the probabilities of both positive and negative health outcomes, with an unbiased professional recommendation for survival duration vs. QoL, best guides pet-parents to optimal decision-making. Be honest about downsides early on. Ask about the pet-parent's preferences and ability for involvement in future care.

### 4.2. Managing progressive symptoms

A second key aspect in palliative care is effective management of progressive symptoms. Disease progression is inevitable in life-limiting disease.

#### 4.2.1. Preparation

Preparing pet-parents by explaining what to look out for and how to manage disease progression is essential to avoid unnecessary emergency visits and hospitalizations. From the beginning, the veterinarian outlined the potential scenarios for Joe's condition. This was helpful both in helping avoid unnecessary emergency visits and maintaining a sense of control. For instance, respiratory distress is a significant risk in advanced lung cancer. To avoid unnecessary (and expensive) hospitalizations, Joe's pet-parents were advised to rent an oxygen concentrator and box in case oxygen support was needed (which it wasn't, until Joe's final week). With that, Joe was looked after at home throughout the illness journey without a single hospitalization. In Joe's case, managing cough and pneumonia were the biggest challenges. Certain anti-tussive medications (e.g., Butorphanol) induce drowsiness, whereas antibiotics for managing pneumonia impair appetite, an issue when maintaining nutrition intake became challenging. With the ongoing and timely support from Joe's veterinarian and the nurses, each of the challenges were handled and the pet-parents were able to maximize Joe's comfort as symptoms emerged.

#### 4.2.2. When?

Another common question is “When?” will the disease progress to the stage that pet-parents have to consider the hardest decision to let go? (6) By June, when the X-ray showed the tumor compressing the bronchi, Joe's veterinarian was asked, “what will happen to Joe as the tumor continues to grow, further compressing the bronchi?” She replied “That would be the time to let go”. The thought of letting him go was devastating, but that conversation was certainly helpful, and also raised the topic of advanced directive.

### 4.3. Advanced directives

Decide beforehand about euthanasia. Why? First, acute illness crises are distressing, decision-making becomes emotive and sub-optimal, and may be regretted later. Second, knowing in advance the plan of action enables practical and emotional preparation for death. Emotional preparation allows pet and parents to share quality time before symptom distress intrudes, which facilitates pet-parent bereavement adjustment. Third, Von Clauswitz's adage “no plan survives first contact with the enemy” applies. Disease trajectories vary and, in Joe's case, he thankfully remained independently active until the last afternoon of his life. Despite anticipating and planning for breathlessness, pain and pneumonia, the pet-parents instead frequently faced nocturnal fever spikes requiring ice packs, and appetite loss. In his last week, Joe's love of eating gradually faded. Finding something he enjoyed was helped by daily variation of his diet. But some days he would not eat, so the Vet nurses called Joe to the clinic, hand-feeding him tinned dogfood, which he always relished! The first weekend of August saw him increasingly dyspnoeic and, despite the Oxygen concentrator, Joe was progressively uncomfortable. After a Sunday night that saw him largely sedated it was clear on Monday morning that he was struggling to breathe. He went outside to pee on awakening and stood sniffing the air between panting. When taken back to his bed he unusually ignored the sounds of breakfast time, and just laid there. Joe almost never barked, but at that moment, he made one clear and loud bark. The pet-parents felt this was his request to go. The Vet team was contacted and made a home visit over their lunchbreak that day. Before the euthanasia infusion syringe was even half empty Joe was still. He chose his time, staying independent to the end.

Accompanying the vet were all the clinic nurses who had helped care for Joe over the years and who had come to say goodbye. To know he was such a widely-loved old boy meant so much to us.

The bereavement was long, and 18 months later we have found a way to both accept Joe is gone while keeping his memory alive. It hasn't always been easy.

Joe enjoyed 22 weeks of good quality, stress-free life, independence and indulgence from when his diagnosis was confirmed. We were pleased to have been able to care for him, spoil him a little, and enjoy his remaining time. This wouldn't have been possible to anything like the same extent without the support of a great veterinary team.

## 5. The vet perspective

Within daily veterinary practice, the need to manage pets with life-limiting disease is a growing problem that all vets face on a regular basis. In particular how do we best support pet-parents to care for elderly and terminally ill pets? Good communication is key. Communication enables planning and predictability, which in turn enhances a sense of control over what can feel like a helpless situation to the owner. As Joe‘s case exemplifies, five main elements helped ensure both the pet‘s physical comfort as well as the pet-parents' mental comfort:

### 5.1. Empathy and decision-making.

#### 5.1.1. Empathy

Empathy is something easier said than done. Often, we see so many sick patients that we forget to show sincere compassion to every owner. This is especially difficult on a busy day rushing between patients. When I disclosed an open diagnosis of Joe possibly suffering from pulmonary neoplasia or lung consolidation, the owners were shocked and tearful. Sometimes a pat on the shoulder better shows one's empathy over words (6).

### 5.2. Take time with client communication

Especially in terminal/incurable disease, schedule the owners for the last morning or evening consult enabling extra time and privacy if needed. In Joe's case, with the radiographs suggestive of probable neoplasia, a pre-lunch appointment was allocated, when no other clients are waiting, instead of breaking the news immediately over the phone (never!) or between other consults. Deliver news in digestible pieces, allowing owners time to accept and digest the situation. Check that they are emotionally ready before presenting more information and options to them. This is the ADA approach in practice (6).

### 5.3. Deliver information in Layman's terms using a gentle tone

Joe's owners have medical backgrounds, making it easier to explain Joe's condition without too much confusion. But for most owners, jargon can confuse. So, lay terms, for example, “lung cancer” instead of “pulmonary neoplasia” should be used wherever possible. Check understanding is correct. After owners have understood the diagnosis, the usual illness trajectory in dogs with lung cancer can then be explained, along with localized vs. metastatic disease, and likely signs the animal may show as the disease progress. This increased predictability benefits owners' sense of control, which helps to reduce their stress and anxiety (6). Recognize cancer is a scary thing to most people, vets included.

### 5.4. Advanced directives: provide options and hope

Engage them in the decision-making process after you have presented them with all the information. Giving realistic hope may sound very contradictory when dealing with an incurable disease, but hope is what keeps us all going. Normally when dealing with cancer or other chronic disease, the owner's first question is: Is it curable? If not, what could be done to slow down the progress and relieve any discomfort? In Joe's case, despite facing Pulmonary Adenocarcinoma, there were options to managing his illness and quality of life: surgery and/or palliative treatment. As veterinarians we have the medical knowledge to inform the owners of different treatment methods and their likely survival times but our most important goal is to present this information with the pros and cons to allow the owner to make an informed decision with all the facts. It is important to let the owners make their own decision informed by your guidance and experience. Avoid forcing your ideals onto them, though, as in human cancer treatment decision-making, a clinician's recommendation can be a helpful proxy for a client's lack of technical knowledge. There is no absolute right or wrong decision.

In Joe's case, Primary Pulmonary Adenocarcinoma, diagnosed by CT scan and histopathology, was displacing the bronchus and Aorta. Surgery was feasible, but significantly invasive with its own risks and complications. Medical treatment including curative and palliative chemotherapy would only retard disease progress and relieve symptoms temporarily, but with significant impact on QoL. After explaining the pros and cons of all the options, the clients were asked what they thought was best for Joe: maximum survival time, or comfort with minimal aggressive treatment? It is very important to listen to what the owners want for their pet and guide them through options that best fit their expectations. Joe's owners chose palliative treatment comprising a combination of symptomatic treatment with conventional medicine, supplemented by traditional Chinese Medicine for coughing and delaying the disease progress.

### 5.5. Continuity of care

Lastly, and most importantly, regular follow-ups support owners emotionally and clinically, and provide peace of mind. In Joe's case, phone call follow-ups (either by me personally or clinic nurses) every 2–3 weeks checked if the owners needed any help or guidance. Once every 1-to-2 months Joe came for a check-up. This involved repeat radiographs to check the size of the carcinoma and a basic blood profile to check for significant leucocytosis suggestive of inflammatory process or secondary infection warranting anti-inflammatories or antibiotics. As the disease approached end stage, guidance of how to gauge quality of life was given to the owners and the topic of when was the time to let go was gently brought in. Information about cremation and other options for managing the body was given in case of sudden death. The owners achieved a clear idea of when to let go and had prepared well for Joe's departure. After Joe passed away, the clinic prepared a sympathy card to show our care, love and sorrow.

To conclude, although our experience and medical knowledge is imperative in keeping the patient comfortable, for me, to successfully handle a case well also involves genuine empathic care for the pet and ensuring good open communication is maintained with the owners.

## 6. Summary

Good communications care facilitates physical care, which in turn, can enhance quality of life for both animal and their humans. While we were lucky with Joe in terms of his illness trajectory, and our own professional backgrounds undoubtedly helped, for most companion animal-human families, potentially different scenarios are not uncommon. Even so, the growing recognition of the need for, and emphasis on clinical communications skills training in medical doctors, show the recognition good communications and careful communications planning facilitates decision-making around difficult topics such as breaking bad news, handling questions, and end-of-life care planning, including advanced directives (6). These have consistently shown to benefit quality of life in patients and their families before, and importantly, after death. There is no reason to think that this will not be the case in veterinary care also.

## Data availability statement

The original contributions presented in the study are included in the article/supplementary material, further inquiries can be directed to the corresponding author.

## Ethics statement

Ethical review and approval was not required for the study of animal participants in accordance with the local legislation and institutional requirements. Written informed consent was obtained from the owners for the participation of their animals in this study.

## Author contributions

WL contributed in conception, drafting, and editing the manuscript. RF and LC contributed in drafting and editing the manuscript. All authors contributed to the article and approved the submitted version.
